# Complement Cascade Proteins Correlate with Fibrosis and Inflammation in Early-Stage Type 1 Diabetic Kidney Disease in the Ins2Akita Mouse Model

**DOI:** 10.3390/ijms25031387

**Published:** 2024-01-23

**Authors:** Aggeliki Tserga, Jean Sébastien Saulnier-Blache, Kostantinos Palamaris, Despoina Pouloudi, Harikleia Gakiopoulou, Jerome Zoidakis, Joost Peter Schanstra, Antonia Vlahou, Manousos Makridakis

**Affiliations:** 1 Biomedical Research Foundation, Academy of Athens, Department of Biotechnology, Soranou Efessiou 4, 11527 Athens, Greece; atserga@bioacademy.gr (A.T.); izoidakis@bioacademy.gr (J.Z.); vlahoua@bioacademy.gr (A.V.); 2 Institut National de la Santé et de la Recherche Médicale (INSERM), UMR1297, Institute of Cardiovascular and Metabolic Disease, 31432 Toulouse, France; jean-sebastien.saulnier-blache@inserm.fr (J.S.S.-B.); joost-peter.schanstra@inserm.fr (J.P.S.); 3 Department of Biology, Université Toulouse III Paul-Sabatier, 31062 Toulouse, France; 4 1st Department of Pathology, School of Medicine, National and Kapodistrian University of Athens, 34400 Athens, Greece; kpalamaris@yahoo.gr (K.P.); dpouloudi@med.uoa.gr (D.P.); charagak28@gmail.com (H.G.); 5 Department of Biology, National and Kapodistrian University of Athens, 15701 Zografou, Greece

**Keywords:** Ins2Akita, diabetes, diabetic kidney disease, proteomics, kidney, LC–MS/MS, biomarker, complement, fibrosis, glomeruli

## Abstract

Diabetic kidney disease (DKD) is characterized by histological changes including fibrosis and inflammation. Evidence supports that DKD is mediated by the innate immune system and more specifically by the complement system. Using Ins2Akita T1D diabetic mice, we studied the connection between the complement cascade, inflammation, and fibrosis in early DKD. Data were extracted from a previously published quantitative-mass-spectrometry-based proteomics analysis of kidney glomeruli of 2 (early DKD) and 4 months (moderately advanced DKD)-old Ins2Akita mice and their controls A Spearman rho correlation analysis of complement- versus inflammation- and fibrosis-related protein expression was performed. A cross-omics validation of the correlation analyses’ results was performed using public-domain transcriptomics datasets (Nephroseq). Tissue sections from 43 patients with DKD were analyzed using immunofluorescence. Among the differentially expressed proteins, the complement cascade proteins C3, C4B, and IGHM were significantly increased in both early and later stages of DKD. Inflammation-related proteins were mainly upregulated in early DKD, and fibrotic proteins were induced in moderately advanced stages of DKD. The abundance of complement proteins with fibrosis- and inflammation-related proteins was mostly positively correlated in early stages of DKD. This was confirmed in seven additional human and mouse transcriptomics DKD datasets. Moreover, C3 and IGHM mRNA levels were found to be negatively correlated with the estimated glomerular filtration rate (range for C3 rs = −0.58 to −0.842 and range for IGHM rs = −0.6 to −0.74) in these datasets. Immunohistology of human kidney biopsies revealed that C3, C1q, and IGM proteins were induced in patients with DKD and were correlated with fibrosis and inflammation. Our study shows for the first time the potential activation of the complement cascade associated with inflammation-mediated kidney fibrosis in the Ins2Akita T1D mouse model. Our findings could provide new perspectives for the treatment of early DKD as well as support the use of Ins2Akita T1D in pre-clinical studies.

## 1. Introduction

Diabetic kidney disease (DKD), the leading cause of end-stage kidney disease (ESKD) worldwide, is caused by prolonged exposure to high glucose levels, represented by type 1 (T1D) [1] and type 2 diabetes (T2D) [2,3]. DKD is characterized by kidney ultra-structural and morphological alterations such as mesangial expansion, nodular glomerular sclerosis, glomerular basement membrane (GBM) thickening, and tubulointerstitial fibrosis [4]. DKD is often clinically detected through increased albuminuria and a decreased glomerular filtration rate [5].

The main disease mediators for DKD are considered metabolic and hemodynamic factors [6] leading ultimately to glomeruli injury, this characterizing the early stages of DKD [7]. DKD progresses slowly and renal biopsy is not routine in patients with diabetes; it is only rarely performed when rapid progression in renal function impairment and severe proteinuria is observed [8]. A substantial amount of data have supported the major role of innate and adaptive immune-mediated inflammation in DKD development and progression [5,9,10,11]. In DKD, hyperglycemia, advanced glycation end products (AGEs), high lipid levels, and increased oxidative stress damage renal cells, leading to increases in both pro-inflammatory signaling pathways and the complement cascade [12,13,14,15]. Eventually, the sustained chronic inflammation leads to an altered kidney structure and fibrosis [9,16,17,18,19,20,21].

The complement system consists of more than 30 proteins. It is part of the first line of defense in innate immunity by clearing the organism from pathogenic microbes and enhancing the removal of immune complexes and apoptotic cells. Although it is part of the innate immune system, it is also recruited by antibodies joining innate and acquired immunity. Complement activation can be initiated by three triggers: the binding of IgG or IgM immune complexes to C1q (classical pathway), the continuous C3 hydrolysis (alternative pathway), and the interaction of mannose-binding lectin (MBL) with bacterial glycosylated molecules rich in mannose (lectin pathway) [15,22].

A transcriptome and immunohistochemical analysis of human kidney biopsies (stages III-IV of DKD) revealed that about 50% of all DKD cases have an increased glomerular deposition of C3 compared to healthy controls, associated with increased glomerulosclerosis [23].

The potential role of the complement system in DKD pathogenesis was further obtained from studies in animal models, detecting the kidney deposition of C3 protein in T1D and T2D. In T1D non-obese diabetic (NOD) mice, OVE26 mice, and Streptozotocin (STZ) diabetic rats, C3 deposition was detected in the early stages of DKD [22]. In T2D KK-Ay mice, C3 was detected in later stages of DKD in glomeruli [22]. However, to our knowledge, there is no study detecting complement activation in the Ins2Akita mouse, even though it is characterized as an excellent T1D mouse model of DKD compared to STZ, NOD, and OVE26 models [24,25,26,27].

In recent years, great efforts have been made in order to develop complement-targeting drugs since complement activation is one of the primary pathogenic mechanisms in several inflammatory diseases [28]. Concerning nephropathies, only Eculizumab, a C5 inhibitor, has FDA approval so far and is currently being tested in C3 glomerulopathies [29]. Other drug inhibitors targeting complement receptors and the lectin pathway are also tested in animals and clinical studies [22]. Moreover, the use of inmmunomodulatory drugs has been found to diminish IgM glomerular deposits and proteinuria in an adriamycin mouse model, and to protect and reverse diabetes in autoimmune cases [30,31] as well as T2D [32].

Given the increasing therapeutic potential of complement inhibitors, our study aimed at shedding more light on the association of complement activation with development of inflammation and fibrosis in early DKD. Using the Ins2Akita mice as the model system, this study also investigated for the first time the overall suitability of this model to reflect these processes in early DKD, opening up new avenues for pre-clinical research in DKD therapy and early biomarker discovery.

## 2. Results

### 2.1. Complement Cascade, Inflammation, and Fibrosis Correlate in DKD in Animal Models and Human Datasets

From the proteomics data of the published study on the Ins2Akita model [33], we observed an upregulation of proteins related to the complement cascade, fibrosis, and inflammation (Table 1). Specifically, C3, C4B, and IGHM were upregulated in glomeruli of 2-month-old mice and C4B and IGHM in glomeruli of 4-month-old mice vs. controls (Table 1). Multiple proteins related to fibrosis were also found to be upregulated in 2-month-olds (including ENG and CORO-1C), 4-month-olds (FLNA and MYOF), or both time points (FN1 and DYSF) (Table 1). Concerning the inflammation pathway, multiple related proteins were detected as upregulated in glomeruli of early-DKD mice including MTDH, PSMD11, CLIC4, TGFb1i1 whereas TSPAN2 and MCAM were upregulated in glomeruli of moderately advanced DKD versus the respective controls (Table 1).

In order to place and validate these findings from the Ins2Akita mice in the context of published datasets regarding DKD, kidney transcriptomics data from patients with DKD and animal models in comparison to respective healthy controls were retrieved from the Nephroseq database. Specifically, seven relevant profiling datasets corresponding to different publications could be retrieved ([23,34,35,36,37]). Data on all, except three proteins (C4B, PSMD11, and YTHDF1), could be retrieved. In parallel, literature mining on these proteins (Table 2) was also performed. This analysis indicated, in most cases, an agreement in the expression trends at the protein and mRNA levels of the investigated molecular features as summarized in Table 2.

### 2.2. Positive Correlation of Expression of Complement Proteins with Fibrosis- and Inflammation-Related Proteins in Ins2Akita Mice

Given the conservation of the observed changes in the animal models and humans, a further investigation of links between the processes of complement activation, fibrosis, and inflammation was performed. As a first step, a Spearman rho correlation analysis was performed for all detected proteins representing the three processes in 2-months-old mice, 4-months-old mice, and both groups combined [33]. As shown in Table 3, a positive and significant correlation of the expression of the three proteins of the complement cascade and fibrosis-related (Table 3 and Table S1) or inflammation-related proteins (Table 4 and Table S2) in early DKD in Ins2Akita mice could be observed.

A Spearman rho analysis was also performed using human transcriptomics data retrieved from the Nephroseq database (five human datasets in total; [23,34,36,37]) and literature data, as available. As shown in Table 5 and Table 6, renal transcriptomics datasets provide data both from glomerular and tubular parts of the kidney. As shown, most of the results are in agreement with our findings from the Ins2Akita model (Table 5 and Table 6) with some cases, however, showing inverse correlation trends. The latter may be attributed to either the representation of mainly advanced DKD samples in Nephroseq and/or differences between mRNA and protein expression patterns. In addition, as shown (Table 5 and Table 6), in a few cases, correlation analysis results were conflicting among the glomerular and tubular data. However, many consistencies can be clearly observed and in addition, the significance of the detected positive correlations among these proteins is enforced by the literature, as it is shown in Table S3 with the majority of our findings being supported in mouse and human studies of several inflammatory diseases.

These results collectively provide strong evidence supporting the positive correlation of expression between complement proteins and fibrosis- and inflammation-related proteins in DKD, also apparently occurring at an early disease stage as represented in the Ins2Akita DKD mouse model.

### 2.3. Validation of Complement Upregulation and Its Association to Fibrosis and Inflammation in Human Patients with DKD

In order to further investigate the correlation of the complement cascade with DKD-stage inflammation and fibrosis, a set of 43 tissue sections of human kidney DKD tissues (class I–IV) were analyzed using immunofluorescence for the expression of C3, C1q, C4, and IGM proteins (immunofluorescence results and demographic characteristics of the patients are shown in Table S4). Histologically, the majority of the cases (n = 23) were classified as class III diabetic kidney disease. The remaining 19 patients were distributed among the other three classes, with 6 classified as class IV, 17 as class II, and 1 case as class I. Regarding biopsies, the chronicity index was involved, median global glomerulosclerosis (GS) was 33.33% (range: 10–80.95%), and the median percentage of renal cortex area occupied by fibrotic tissue and atrophic tubules was 35% (range: 15–70%). In general, higher levels of chronicity, both glomerular and tubulointerstitial, were observed in classes III and IV, as in class IV, GS is by definition over 50% of the sample’s glomeruli. Moreover, extensive hyalinoses, segmental scleroses, as well as hyaline drops and fibrin caps were encountered in biopsies with advanced stages. Immunofluorescence revealed the deposition of complement factors C3, C1q, and C4, as well as of IgM with heterogenous distribution. The most frequently encountered complement protein was C3, which was detected in 22 biopsies (Table 7).

**Figure 1:**
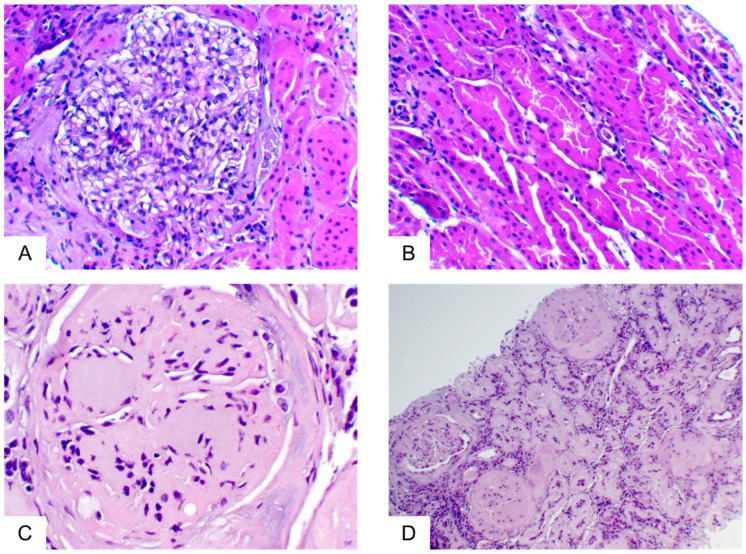
Both glomerular and tubulointerstitial compartments displayed no significant alterations in a biopsy from a class I DKD case (**A**,**B**: H&E stain, 200×), while in a specimen of class IV, DKD glomeruli demonstrated multiple clearly formed Kimmelstiel–Wilson nodules (**C**: H&E stain, 400×) and tubulointerstitial tissue shows extensive tubular atrophy, accompanied by areas of fibrosis and multifocal inflammatory infiltrates (**D**: H&E stain, 100×).

**Figure 2:**
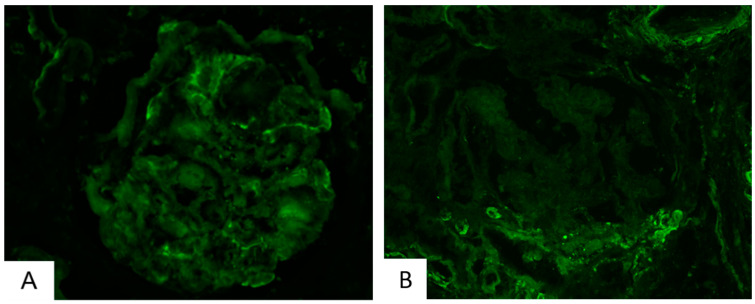
Immunofluorescence of C3 revealed glomerular deposits in a biopsy with class IV (**A**) (400×) and class II (**B**) (200×) DKD.

**Figure 3:**
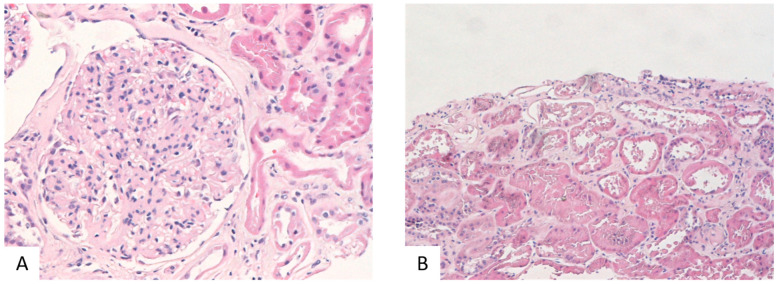
In renal biopsy from a class II DKD, glomeruli were characterized by increased size, with mild to moderate expansion of mesangial matrix (**A**: H&E stain, 200×). Tubulointerstitial compartment displayed mild tubular atrophy and interstitial fibrosis (**B**: H&E stain, 200×).

Interestingly, three specimens (class III), characterized by C4 staining, were also positive for C1q. Moreover, in sixteen specimens, the prevalences of glomerular IGM deposits were significantly correlated with these of glomerular C3 deposits (*p* < 0.001). In general, all complement factors examined, as well as IgM molecules, were located almost exclusively in glomeruli, primarily in areas of hyalinosis and segmental scleroses, where they could represent non-specific aggregates. In one case, C3 deposits were restricted in tubules, while in three biopsies, C1q tubular staining accompanied glomerular staining. Regarding the correlation of complement or IgM deposits with clinicopathological parameters, C3 displayed significant correlation with a higher IFTA (interstitial fibrosis/tubular atrophy) (*p* = 0.027) score and GS (glomerulosclerosis) (*p* = 0.0048), as well as with more extensive areas of tubular atrophy (*p* = 0.008) and interstitial fibrosis (*p* = 0.024). C1q was correlated with a higher fibrosis index (*p* = 0.012) and with more severe impairment of renal function, as defined by a decrease in the glomerular filtration rate (eGFR) (*p* = 0.03). Overall, DKD was statistically significant correlated with C3 (*p* = 0.001) and IGM (*p* = 0.038)-positive staining. Our findings concerning the immunofluorescence analysis of the human kidney further confirmed the abundance of complement-related (C3, C1q, and IGM) proteins in DKD and their correlation with fibrosis and inflammation mainly in advanced DKD (Figure 1 and Figure 2A). Three samples out of the sixteen samples of class II were positive in C3 IF staining; thus, occasionally, C3 is detected from an early timepoint in disease development in humans, similar to the Ins2Akita mice; more samples have to be analyzed to find in more detail the pattern of C3 expression in early human DKD (Figure 2B). Mild fibrosis is also detected in early-DKD biopsies (Figure 3).

**Figure 4:**
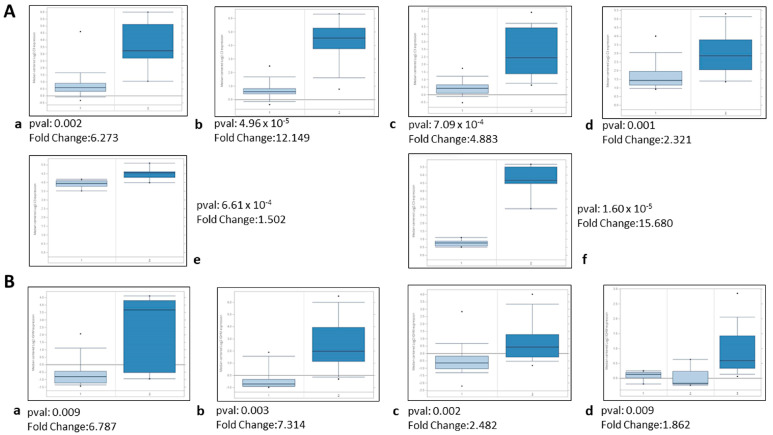
The expression of C3 and IGHM in DKD samples when compared with healthy control tissues in human and mouse transcriptomics datasets as per availability (source Nephroseq). (**A**) The expression of C3 in DKD renal tissues is higher than in healthy control renal tissues in human (**a**) Woroniecka Diabetes Glom GSE30528 [23], (**b**) Woroniecka Diabetes TubInt GSE30529 [23], (**c**) Ju CKD Glom GSE47183 [36], and (**d**) Ju CKD Tubuli GSE47184 [37] datasets and mouse (**e**) Hodgin Diabetes DBA Mouse Glom GSE33744 [35] and (**f**) Hodgin Diabetes Mouse Glom eNOS-deficient C57BLKS db/db GSE33744 [35] datasets. (**B**) The expression of IGHM in DKD renal tissues is higher than in healthy control renal tissues in human (**a**) Woroniecka Diabetes Glom GSE30528 [23], (**b**) Woroniecka Diabetes TubInt GSE30529 [23], (**c**) Ju CKD Tubuli GSE47184 [37], and (**d**) S = Schmid Diabetes TubInt [34] datasets. 1 = Healthy controls, 2 = DKD samples (concerning Figure **B**(**d**): 1 = Cadaveric donor controls, 2 = Healthy controls, and 3 = DKD samples).

In order to further study the association of the complement cascade with DKD progression, we performed an in silico analysis with published transcriptomics data. Of the three complement-related proteins differentially expressed in our glomerular dataset of Ins2Akita mice, transcriptomics data of C3 and IGHM could be retrieved. With the use of Nephroseq v5, the expression of C3 and IGHM genes showed the difference between patients with DKD and animal models and respective healthy controls (Figure 4); we detected these two genes as upregulated in the renal tissues of DKD samples vs. the healthy kidney tissues. The correlation analysis of the expression of these two complement proteins (C3 and IGHM) from the Nephroseq datasets retrieved positive statistically significant results (Woroniecka Diabetes TubInt GSE30529 [23] *p* = 0.0001, r = 0.735; Woroniecka Diabetes Glom GSE30528 [23] *p* = 0.0002, rs = 0.706; Ju CKD TubInt GSE47184 [37] *p* = 0, rs = 0.46). In addition, in order to validate the potential role of C3 and IGHM in renal function, a correlation analysis of these two genes and eGFR of patients with DKD was conducted using the Nephroseq v5 online tool (Figure S1). C3 (Woroniecka Diabetes Glom GSE30528 [23] *p* = 0.005, rs = −0.580; Woroniecka Diabetes TubInt GSE30529 [23] *p* = 8.95 × 10^−7^, rs = −0.842; Schmid Diabetes TubInt [34] *p*= 0.046, rs = −0.611) and IGHM (Woroniecka Diabetes Glom GSE30528 [23] *p* = 0.003, rs = −0.604 and Woroniecka Diabetes TubInt GSE30529 [23] *p* = 8.15 × 10^−5^, rs = −0.740) mRNA levels in renal tubular and glomerular tissue were negatively correlated with eGFR in human patients with DKD, suggesting that increased C3 and IGHM expression is linked to reduced renal function.

## 3. Discussion

An early diagnosis of DKD could increase the quality and duration of life of patients with diabetes through the delay of kidney failure. In-depth understanding of the molecular mechanisms of DKD could assist in its early detection.

Within this context, accumulating evidence demonstrates that DKD is progressing with a variety of mechanisms, including immune and inflammatory processes [57,58]. The complement cascade is a central part of innate immunity; however, its hyper-activation leads to systemic diseases with apparent enhanced inflammation and fibrosis [5,12]. A recent integrative study of five relevant gene datasets predicted the status of immune cell infiltration and immune-related biomarkers in DKD using bioinformatic approaches. FN1 and C3 were found to be closely related to the pathogenesis and progression of DKD, as well as macrophage infiltration [59]. Complement activation could contribute to the inflammatory environment in DKD, and thus be linked with fibrosis, which leads to progressive loss of kidney function [12]. Despite the existent supporting evidence, further work is required to define the role of the complement system in DKD progression.

The first evidence correlating the complement system in DKD development was provided by the finding that in serum, urine, and renal samples from patients with diabetes, complement proteins were detected and associated with DKD [12,23,60,61,62,63]. Moreover, elevated plasma levels of C3 were detected in patients with T2D and macroalbuminuria in comparison to those with normoalbuminuria [38]. In a large-scale cohort study, high plasma levels of C3 of patients with diabetes were associated with potential kidney damage [39]. Furthermore, it is suggested that C3 serum levels could differentiate patients with DKD from patients with diabetes without kidney damage [40].

The current study was set out with the scope to increase understanding of the complement role and connection with inflammation and fibrosis in the Ins2Akita model of early DKD, and also to assess its validity as a pre-clinical model of DKD. This model is considered representative of T1D; nevertheless, complement activation in the context of early DKD has not been demonstrated. Our study was mainly focused on renal proteins that were upregulated in T1D animals in early (2 months) and moderately advanced (4 months) DKD. We detected three complement-pathway-related proteins (C3, C4B, and IGHM) as upregulated in early (2 months)-DKD InS2Akita mice. Among these proteins, C3 is the central component of the complement pathway and has a major role in the classic and alternative complement cascade [64]. Most of the inflammation-related proteins are upregulated in early DKD; however, most fibrotic-related proteins are induced in later stages of DKD. It is known that inflammation has a pivotal role in the initiation of DKD through the increased levels of both pro-inflammatory signaling pathways and the complement [12,13,14,15]. The innate immune system (a member of which is the complement cascade) is one of the major driving factors in the inflammatory response in DKD [13]. The activation of the inflammatory response eventually leads to cell injury and development of kidney fibrosis [9,16,17,18,19,20,21].

We performed correlation analyses of the expression of complement proteins versus inflammation- and fibrosis-related proteins that were statistically significant changed (upregulated—Table 1) and in parallel with the whole Ins2Akita protein dataset. This study shows for the first time the parallel abundance and mainly positive association of the complement cascade, fibrosis, and inflammation in the Ins2Akita mouse (Table 3 and Table 4). Most of the correlations were observed in mice of 4 months of age and upon combination of 2- and 4-months-of-age datasets, the latter likely linked to the higher statistical power (sample size) of the analysis. Via the correlation and importantly the cross-correlation analysis with the Nephroseq datasets and the literature, collectively, our study highlights and brings together multiple protein changes in the complement cascade, inflammation, and fibrosis, which had to a good extent been observed at the mRNA level in animal models or human tissue of mainly advanced DKD stages, supporting, as a step further, their role at an early time point in DKD development. 

Besides this in silico analysis, experimental evidence for the correlation of the complement with DKD was also provided via renal histopathology of patients with DKD. C3, as investigated using immunofluorescence, showed the most frequent, among the other complement proteins, kidney deposits and was correlated with fibrosis. C1q was also correlated with fibrosis and the rate of renal function decline as previously shown by Sun et al. and Jiao et al. [65,66]. Overall, C3 and IGM deposits were correlated with DKD, emphasizing the potential role of local complement activation in DKD. Previous studies have also shown the association of complement cascade activation and the pathogenesis of DKD [62,67,68]: Bus et al. showed positive association between renal C1q deposits in glomerular hili and arterioles with DKD occurrence [62]. Pelletier et al. [67] observed that the renal expression of C1q was strongly correlated with inflammation, fibrosis, proteinuria, and the rate of renal function decline in patients with DKD. Morigi et al. [68] suggested that in BTBR ob/ob mice, a diabetes type II model, kidney C3 deposition, and increased renal expression of the C3a and C3a receptor could lead to the development of albuminuria, glomerular injury, and podocyte loss. Increased levels of glomerular complement proteins (including C3 and C4B) were detected in a proteomic analysis from a laser-captured microdissection of human DKD biopsies [41,42]. Moreover, several inflammatory differentially expressed genes were detected in glomeruli of DKD mice by using single-cell RNA sequencing [58].

As early-DKD samples are not regularly available, complement activation in early DKD is not widely studied in human and animal studies. Clinical information of the patients not being available is a limitation. In our study, immunofluorescence staining from stage II DKD samples show a slight activation of the C3 component (Figure 3); however, due to the limited number of human biopsies of early-DKD samples, we could not reach statistically significant results. In a recent study of complement activation in DKD [62], C1q, C4d, and C5b-9 complement deposits were detected in the glomeruli of patients with early DKD. C4c glomerular depositions were detected in early CKD [69]. IGM renal staining was also observed in patients with early DKD [48]. In urine samples, C3 and C4B proteins were detected as upregulated in early-DKD cases [70,71,72] and C6 was detected as upregulated in children with T1D < 1 y in comparison to healthy age-matched children [73]. The results from our study clearly indicate that the Ins2Akita mouse model apparently nicely recapitulates the abundant literature on complement activation and DKD and the few studies on early DKD.

Presently, there is increasing interest in the development of therapeutic compounds of the complement cascade [12,74]. Some molecules are already in clinical use for diseases other than DKD, and these should be tested for their ability to slow or halt the progression of diabetic nephropathy [22]. It is supported from pre-clinical studies that complement repression prevents DKD progression, and targeting of the complement receptor could be a promising therapeutic strategy [22], as well as the use of antibodies against IgM and endoglin [30,31,32,51].

## 4. Materials and Methods

### 4.1. Samples, Glomeruli Preparation, and Biochemical Analysis

The mouse model C57BL/6-Ins2Akita/J (Ins2Akita-T1D) was used. In the glomerular proteome study [33] 4 groups of animals were included: 2-month-old Ins2Akita (INS2) (n = 8) and respective controls (WT2; n = 7), and 4-month-old Ins2Akita (INS4) (n = 8) and respective controls (WT4, n = 8) [33].

Glomeruli were isolated; the murine kidney histology and biochemical analysis were performed as described in [7], Klein J., et al. (2020).

### 4.2. Sample Preparation and LC-MS/MS Analysis and MS Data Processing

Sample preparation, LC-MS/MS, and MS data processing were performed as described in [33], Tserga A., et al. (2022). Samples (200 μg of total protein per sample) were processed with the filter-aided sample preparation (FASP) method as described previously.

### 4.3. Statistical Analysis

The graphing and statistical analysis were performed in the RStudio environment (R version 4.0.3). The direction of protein co-expression (positive/negative) was determined using Spearman’s Rank correlation coefficient and significance was defined at *p* ≤ 0.05. Pearson’s correlation analysis between complement-related gene expression and clinical features (eGFR) in patients with DKD was performed using the Nephroseq v5 online tool (http://v5.nephroseq.org, accessed on 1 August 2023). Results of the statistical analysis between Ins2Akita mice and healthy controls were taken from [33], Tserga A. et al. (2022). Significance was defined at *p* < 0.05 with the non-parametric Mann–Whitney test.

### 4.4. Cross-Correlation to Transcriptomics Data

Nephroseq (www.nephroseq.org, accessed on 1 August 2023) was employed for the investigation of the expression of the shortlisted complement and fibrotic proteins (based on biological relevance) in existing mouse and human transcriptomics datasets. The list of proteins was uploaded in Nephroseq v5 in the form of EntrezGene IDs. DKD dataset selection was held after the application of the following filters: Primary Filters > Group > Diabetic Nephropathy. The corresponding gene expression was searched for in 7 available DKD mouse and human datasets observed after filtering, on the comparison of DKD vs. Healthy Living Donor groups (Table 8).

The characterization of “fibrosis”- and “inflammation”-related proteins (Table 1) is based on the literature and Gene Ontology Biological Process (GOBP) terms from the Molecular Signatures Database (MSigDB) mouse database.

### 4.5. Clinical Material and Immunofluorescence

Forty-three anonymized kidney biopsies diagnosed as DKD (class I–IV), on the basis of suggestive clinical and pathological features, were provided by the human Renal Biopsies archive of the 1st Department of Pathology of Athens (National and Kapodistrian University of Athens, Medical School, Greece; Prof. Gakiopoulou) under ethics-approved protocols (approval number 38, 19 November 2018). The study was approved by the National and Kapodistrian University of Athens School of Medicine Ethics Committee. Due to the retrospective nature of the study, the need for informed consent was waived and a policy of strict anonymity was assured. All patients presented with various renal manifestations, including hematuria, proteinuria, nephrotic syndrome, and the deterioration of renal function. Proteinuria was defined as 24 h urine protein higher than 150 mg. Nephrotic syndrome was defined as nephrotic-range proteinuria (>3.5 gr/24 h urine protein), hypoalbuminemia, dyslipidemia, and peripheral edema. Impairment of renal function was determined based on the estimated glomerular filtration rate (eGFR). Renal biopsy specimens were processed and evaluated using light and immunofluorescence microscopy. Light microscopy assessment was based on Hematoxylin and Eosin (H&E), as well as on additional histochemical staining (PAS, Silver, Masson), conducted on sections derived from paraffin-embedded tissues. The glomerular compartment was classified as class I–IV, according to the presence of an increased mesangial matrix (nodular glomerulosclerosis) and diffuse glomerular basement membrane thickening, as well as the percentage of global glomerulosclerosis (GS). Class I included isolated glomerular basement membrane thickening and only mild, non-specific light microscopy changes that did not fulfil the criteria of classes II–IV. Class II included mild (IIa) or severe (IIb) mesangial expansion that did not fulfil the criteria for class III or IV. Class III included nodular sclerosis, defined as the presence of at least one convincing Kimmelstiel–Wilson lesion and ≤50% GS. Class IV included advanced DN, defined as >50% GS. Interstitial fibrosis and tubular atrophy (IFTA) were scored semi-quantitatively, based on the proportion of the tubulointerstitial compartment affected (0: none, 1: <25%, 2: 25–50%, 3: >50%), as was interstitial inflammation. Direct immunofluorescence was also conducted in sections generated from OCT-embedded frozen tissues, for complement components (including C1q, C3, and C4) and for IgM. The following antibodies were used: polyclonal rabbit anti-human C1q/FITC (Dako Denmark A/S, Glostrup, Denmark/product code: F0254/dilution of 1:50), polyclonal rabbit anti-human C3c/FITC (Dako Denmark A/S, Glostrup, Denmark/product code: F0201/dilution of 1:50), polyclonal rabbit anti-human C4c (Dako Denmark A/S, Glostrup, Denmark/product code: F0169/dilution of 1:50), and polyclonal rabbit anti-human IgM/FITC (Dako Denmark A/S, Glostrup, Denmark/product code: F0203/dilution of 1:50). Staining intensity in each renal tissue section was semi-quantitatively graded on a scale of 0–3. Semi-quantification was performed based on the intensity of the immunofluorescence staining. Both morphological evaluations, based on histochemical stains and qualitative immunofluorescence assessment, were conducted by two independent pathologists (H.G. and K.P.) blinded to clinical data and with complete interobserver compliance.

## 5. Conclusions

To our knowledge, our results suggest for the first time that Ins2Akita mice are a diabetic mouse model with a significant abundance of the complement cascade contributing to inflammation-mediated kidney fibrosis, inflammation, and damage in early T1D DKD. The Ins2Akita mouse could, therefore, be a good pre-clinical model to study complement and inflammation inhibition and might provide novel, more specific therapeutic targets for the treatment and early detection of DKD. Unravelling the immune system role in early DKD could allow the use of drugs such as complement inhibitors to treat DKD even in early stages.

## Figures and Tables

**Table 1:** Statistically significant protein changes related to complement, fibrosis, and inflammation between Ins2Akita mice of 2 and 4 months and the respective WT control mice (from previous published data 33).

**COMPLEMENT**					
**Protein**	**Protein Name**	**MW_p_INS2vsWT2**	**Ratio_INS2vsWT2**	**MW_p_INS4vsWT4** (ns = not significant)	**Ratio_INS4vsWT4**
**C3**	Complement C3	**0.001**	**5.073**	0.058 (ns)	1.885
**C4B**	Complement C4-B	**0.009**	**5.568**	**0.003**	**4.425**
**IGHM**	Ig mu chain C region	**0.023**	**4.941**	**0.001**	**13.972**
**FIBROSIS**					
**Protein**	**Protein Name**	**MW_p_INS2vsWT2**	**Ratio_INS2vsWT2** (ns = not significant)	**MW_p_INS4vsWT4**	**Ratio_INS4vsWT4**
**FN1**	Fibronectin	**0.0292**	**3.047**	**0.0005**	**103.182**
**ENG**	Endoglin	**0.028**	**6.629**	0.525 (ns)	1.276
**DYSF**	Dysferlin	**0.032**	**2.156**	**0.004**	**4.548**
**CORO-1C**	Coronin-1C	**0.04**	**1.846**	0.382 (ns)	1.444
**FLNA**	Filamin-A	0.694 (ns)	1.172	**0.0003**	**2.612**
**MYOF**	Myoferlin	0.463 (ns)	1.176	**0.0006**	**2.685**
**INFLAMMATION**					
**Protein**	**Protein Name**	**MW_p_INS2vsWT2**	**Ratio_INS2vsWT2**	**MW_p_INS4vsWT4** (ns = not significant)	**Ratio_INS4vsWT4**
**PSMD11**	26S proteasome non-ATPase regulatory subunit 11	**0.008**	**3.654**	0.792 (ns)	0.863
**IFITM3**	Interferon-induced transmembrane protein 3	**0.038**	**6.487**	0.160 (ns)	2.231
**TGFb1i1**	Transforming growth factor beta-1-induced transcript 1	**0.029**	**3.777**	0.072 (ns)	1.967
**CLIC4**	Chloride intracellular channel protein 4	**0.040**	**1.702**	0.645 (ns)	1.405
**ECSIT**	Evolutionarily conserved signaling intermediate in Toll pathway	**0.042**	**3.356**	0.562 (ns)	0.811
**MTDH**	Protein LYRIC	**0.043**	**25.173**	0.653 (ns)	0.743
**YTHDF1**	YTH domain-containing family protein 1	**0.044**	**3.788**	0.381 (ns)	0
**MCAM**	Cell surface glycoprotein MUC18	0.596 (ns)	1.611	**0.005**	**7.291**
**TSPAN2**	Tetraspanin-2	0.08 (ns)	4.429	**0.004**	**3.465**

**Table 2:** Cross-omics validation of complement-cascade-, inflammation-, and fibrosis-related proteins differentially expressed in early DKD in the Ins2AKITA model, and in human and other DKD models (in all cases, comparisons versus healthy or wild-type controls were performed; the green color denotes increase and the red color denotes decrease in the respective protein levels in Ins2Akita model vs. controls).

Protein	Transcriptomics Expression (Nephroseq; in DKD vs. Controls) [Ref.]	Protein Expression [Ref.]
**Complement**		
**C3**	**increased** (human DKD tubuli and glomeruli [23,36,37] and human tubuli ERCB) **increased** (mouse db/db glomeruli [35])	**increased** (human T2D plasma [38], human DKD plasma [39], human T2D DKD serum [40], human T2D DKD glomeruli [41,42]) **increased** (rat/mouse T1D glomeruli [43,44,45,46], mouse T2D glomeruli [11], rat T2D DKD tubuli [47])
**IGHM**	**increased** (human DKD tubuli and glomeruli [23], human T2D tubuli [36,37], and human tubuli ERCB)	**increased** (human T2D DKD glomeruli [48,49]) **increased** (mouse T1D DKD [44], mouse T1D DKD glomeruli [46], mouse T2D DKD glomeruli [11])
**Fibrosis**		
**FN1**	**increased** (human DKD tubuli and glomeruli [23,34,36] and human tubuli ERCB) **increased** (mouse db/db glomeruli [35])	**increased** (human T2D DKD glomeruli [42]) **increased** (mouse T2D kidney [49], mouse T2D kidney cortex [50])
**ENG**	**increased** (human tubuli ERCB) decreased (human DKD glomeruli [23])	**increased** (human T2D DKD kidney [51]) **increased** (mouse T1D DKD glomeruli [52])
**DYSF**	**increased** (mouse db/db glomeruli [35])	
**CORO-1C**	**increased** (human DKD glomeruli [23], human DKD glomeruli [36])	
**FLNA**	**increased** (human tubuli ERCB) **increased** (mouse db/db glomeruli [35])	**increased** (human DKD glomeruli [42])
**MYOF**	**increased** (human DKD tubuli [23,34,37]—tubuli and human tubuli ERCB) decreased (human DKD glomeruli [23]) **increased** (mouse db/db glomeruli [35])	
**Inflammation**		
**ECSIT**	**increased** (mouse db/db glomeruli [35])	
**MTDH**	**increased** (human DKD tubuli [23]) decreased (human DKD glomeruli [23], human DKD tubuli [34])	**increased** (mouse db/db [53])
**PSMD11**		**increased** (glomerular mesangial cells (GMCs) under high-glucose condition [54])
**MCAM**	**increased** (human DKD tubuli [23], human DKD glomeruli [36], and human tubuli ERCB) **increased** (mouse db/db glomeruli [35])	**increased** (human T2D DKD kidney [55] and human DKD [56]) **increased** (mouse db/db [56])
**TSPAN2**	decreased (human DKD glomeruli [23], human DKD glomeruli [36], and human tubuli ERCB) **increased** (mouse db/db glomeruli [35])	
**IFITM3**	**increased** (human DKD tubuli [23] and human tubuli ERCB)	
**TGFB1i1**	**increased** (human DKD tubuli [23], human tubuli ERCB, mouse db/db glomeruli [37]) decreased (human DKD glomeruli [23])	
**CLIC4**	**increased** (human DKD tubuli [34]) decreased (human DKD tubuli [23])	

**Table 3:** Results of the Spearman rho correlation analysis of expression of the complement proteins vs. fibrosis-related proteins performed in mice of 2 months of age, 4 months of age, and the two groups combined. Statistically significant correlations are shown with asterisks, *** *p* < 0.001; ** *p* < 0.01; * *p* < 0.05. (ns = not significant).

	Complement Cascade								
	Ins2Akita, 2 Months			Ins2Akita, 4 Months			Ins2Akita, 2 and 4 Months		
Fibrosis-Related Proteins	C3	C4B	IGHM	C3	C4B	IGHM	C3	C4B	IGHM
**ABAT**	**	ns	***	ns	ns	ns	*	ns	*
**FN1**	ns	ns	ns	***	*	ns	ns	ns	**
**ARF6**	ns	ns	ns	ns	*	ns	ns	ns	ns
**STXBP1**	ns	ns	ns	ns	***	ns	ns	ns	*
**FLNA**	ns	ns	ns	ns	*	ns	*	***	***
**CYFIP1**	ns	ns	ns	ns	*	ns	ns	ns	ns
**MYOF**	*	*	**	ns	**	ns	*	**	***
**IQGAP1**	ns	ns	ns	ns	*	ns	ns	ns	ns
**ITGB1**	ns	ns	ns	ns	*	ns	ns	*	**
**ITGA1**	*	ns	**	ns	ns	ns	**	*	**

**Table 4:** Results of the Spearman rho correlation analysis of expression of the complement proteins vs. inflammation-related proteins performed between mice of 2 months of age, 4 months of age, and the two groups combined. Statistically significant correlations are shown with asterisks, ** p < 0.01; * p < 0.05. (ns = not significant).

	Complement Cascade								
	Ins2Akita, 2 Months			Ins2Akita, 4 Months			Ins2Akita, 2 and 4 Months		
Inflammation-Related Proteins	C3	C4B	IGHM	C3	C4B	IGHM	C3	C4B	IGHM
**CD81**	ns	ns	ns	ns	ns	ns	*	*	**
**ICAM1**	ns	ns	ns	ns	*	ns	ns	*	**
**VNN1**	*	*	ns	ns	ns	ns	*	ns	ns
**SNAP23**	ns	ns	*	ns	**	ns	*	*	**
**HSPD1**	*	ns	**	ns	ns	ns	ns	ns	ns
**TSPAN2**	*	*	ns	ns	*	ns	ns	ns	ns

**Table 5:** Correlation analysis of the expression of complement proteins vs. fibrosis-related proteins detected in the Nephroseq human datasets. Statistically significant correlations are shown. Positive correlations are highlighted in green and negative correlations are highlighted in red. The complement proteins with statistically significant correlation of expression with fibrosis-related proteins are shown in parentheses. Two glomerular datasets were considered (J = Ju CKD Glom GSE47183 [36], W = Woroniecka Diabetes Glom GSE30528 [23]), and three tubuli datasets (J = Ju CKD Tubuli GSE47184 [37], S = Schmid Diabetes TubInt [34], and W = Woroniecka Diabetes TubInt GSE30529 [23]), as per availability (each reference is shown in parentheses).

	Complement Cascade	
Fibrosis-Related Proteins	Nephroseq Glomeruli	Nephroseq Tubuli
**ABAT**		**rs = −0.791, *p* = 0.001 (C3, 37)**
**FN1**	**rs = 0.802, *p* = 0.001 (C3, 36), rs = 0.723, *p* = 0.003 (IGHM, 36)**	**rs = 0.733, *p* = 0.016 (IGHM, 23), rs = 0.571, *p* = 0.041 (C3, 34)**
**ARF6**	**rs = −0.631, *p* = 0.016 (IGHM, 36)**	**rs = 0.63736, *p* = 0.01912 (C3, 37)**
**STXBP1**	**rs = −0.569, *p* = 0.034 (IGHM, 36)**	**rs = 0.615, *p* = 0.025 (C3, 34)**
	**rs = 0.667, *p* = 0.049 (C3, 23)**	
**FLNA**		**rs = 0.571, *p* = 0.041 (C3, 37)**
**CYFIP1**		**rs = 0.78, *p* = 0.002 (C3, 37), rs = 0.745, *p* = 0.013 (C3, 23)**
**MYOF**		**rs = 0.747, *p* = 0.003 (C3, 37)**
**IQGAP1**		**rs = 0.758, *p* = 0.003 (C3, 37), rs = 0.758, *p* = 0.011 (C3, 18)**
**ITGB1**		**rs = 0.906, *p* = 2 × 10^−5^ (C3, 34)**

**Table 6:** Correlation analysis of the expression of complement proteins vs. inflammation-related proteins detected in the Nephroseq human datasets. Statistically significant correlations are shown. Positive correlations are highlighted in green and negative correlations are highlighted in red. The complement proteins with statistically significant correlation of expression with fibrosis-related proteins are shown in parentheses. Two glomerular datasets were considered (J = Ju CKD Glom GSE47183 [36], W = Woroniecka Diabetes Glom GSE30528 [23]), and three tubuli datasets (J = Ju CKD Tubuli GSE47184 [37], S = Schmid Diabetes TubInt [34], and W = Woroniecka Diabetes TubInt GSE30529 [23]), as per availability (each reference is shown in parentheses).

	Complement Cascade	
Inflammation-Related Proteins	Nephroseq Glomeruli	Nephroseq Tubuli
**CD81**	**rs = −0.556, *p* = 0.039 (C3, 36)**	**rs = 0.852, *p* = 0.00022 (C3, 37),** **rs = 0.555, *p* = 0.049 (IGHM, 37), rs = 0.842, *p* = 0.0003 (C3, 34)**
**ICAM1**		**rs = 0.56, *p* = 0.046 (C3, 37), rs = 0.692, *p* = 0.009 (C3, 34)**
**VNN1**		**rs = −0.632, *p* = 0.02 (C3, 37)**
**TSPAN2**	**rs = −0.543, *p* = 0.044 (C3, 36)**	
**SNAP23**		**rs = 0.733, *p* = 0.016 (C3, 23)**

**Table 7:** Complement deposition according to DKD classification and pathological characteristics of patients and association with fibrosis. Evaluation of chronicity index in renal biopsies was conducted according to the IFTA system, which includes the combination of the percentage of tubular atrophy (TA) and interstitial fibrosis (IF). (The *p* values with statistical significance, 2-tailed, *p* ≤ 0.05, are marked in bold.)

	Glomerular C3 Deposition	Glomerular C1q Deposition	Glomerular C4 Deposition	Glomerular IgM Deposition
**DKD class**				
I	0/1	0/1	0/1	1/1
II	3/17	0/17	0/17	3/17
III	14/19	3/19	3/19	9/19
IV	5/6	2/6	0/6	5/6
**Fibrosis**	**rs = 0.343, *p* = 0.024**	**rs = 0.379, *p* = 0.012**	rs = 0.21264, *p* = 0.171	rs = 0.17478, *p* = 0.2623
**eGFR**	rs = −0.05, *p* = 0.7597	**rs = −0.348, *p* = 0.03**	rs = −0.154, *p* = 0.349	rs = −0.118, *p* = 0.472
**IFTA**	**rs = 0.337, *p* = 0.027**	rs = 0.245, *p* = 0.113	rs = −0.028, *p* = 0.858	rs = 0.082, *p* = 0.598
**GS**	**rs = 0.422, *p* = 0.0048**	rs = 0.163, *p* = 0.294	rs = −0.018, *p* = 0.907	rs = 0.174, *p* = 0.264
**Tubular atrophy**	**rs = 0.399, *p* = 0.008**	rs = 0.282, *p* = 0.067	rs = 0.089, *p* = 0.567	rs = 0.095, *p* = 0.543

**Table 8:** Summary of DKD datasets extracted from Nephroseq database.

Study/Set/Reference	Group
Schmid Diabetes/Tubulointerstitium/[34]	DKD vs. Pretransplant kidney donors
Hodgin Diabetes Mouse/Glomerulus/[35]	DKD mouse model vs. Non-DKD
Ju CKD/Glomerulus/[36]	DKD human vs. Healthy
Ju CKD/Tubulointerstitium/[37]	DKD human vs. Healthy
Woroniecka Diabetes/Tubulointerstitium/[23]	DKD human vs. Healthy
Woroniecka Diabetes/Glomerulus/[23]	DKD human vs. Healthy
ERCB Nephrotic Syndrome/Tubulointerstitium (unpublished study; there are data available only in Nephroseq)	DKD human vs. Healthy

## Data Availability

All data are available in the main text and the supplemental files.

## Supplementary Materials

The following supporting information can be downloaded at: https://www.mdpi.com/article/10.3390/ijms25031387/s1. References [74,75,76,77,78,79,80,81,82,83,84,85,86,87,88,89,90,91,92,93,94,95,96,97,98,99,100] are cited in the supplementary.
