# Supplementary material for: Complement Cascade Proteins Correlate with Fibrosis and Inflammation in Early-Stage Type 1 Diabetic Kidney Disease in the Ins2Akita Mouse Model
Source: Int J Mol Sci. 2024 Jan 23;25(3):1387. doi: 10.3390/ijms25031387 (PMC10855735; doi:10.3390/ijms25031387)
Supplement: Supplementary file 1 [file ijms-25-01387-s001.zip › Figure S1.pdf]

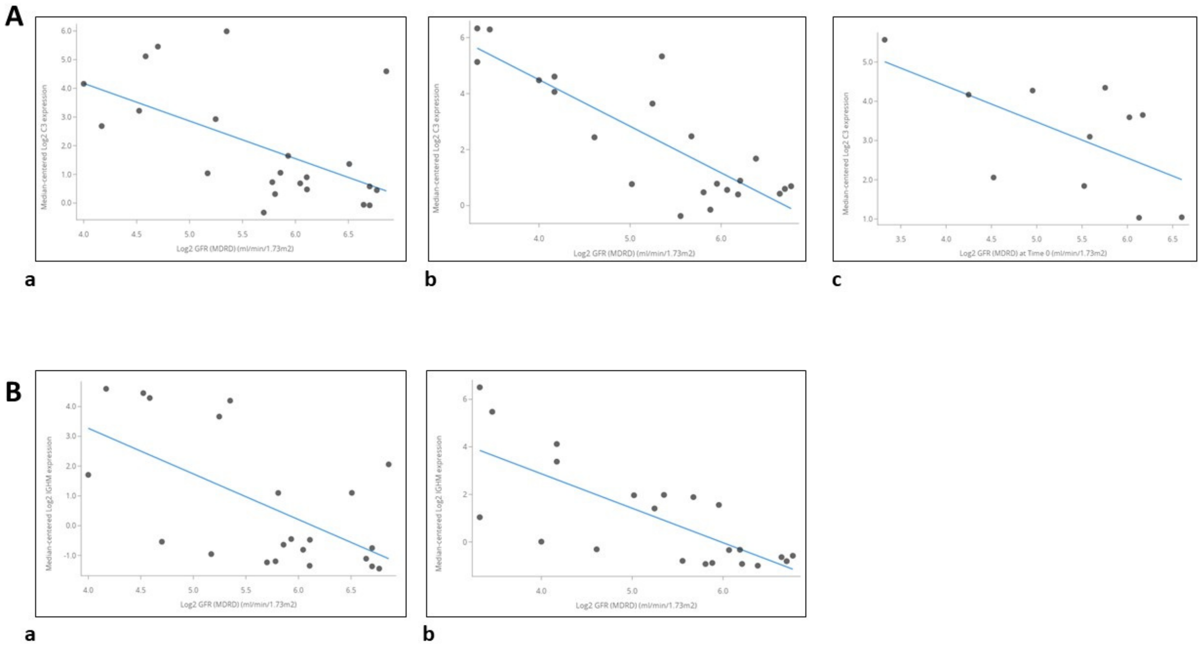

Supplementary Figure S1. The correlation analysis between mRNA expression of C3 and IGHM and eGFR in DN patients as per availability (source Nephroseq). (A) The mRNA expression of C3 was negatively correlated with GFR in 3 datasets: (a) Woroniecka Diabetes Glom GSE30528 [23] p= 0.005, rs= -0.580, (b) Woroniecka Diabetes TubInt GSE30529 [23] p= 8.95e-7, rs= -0.842, (c) Schmid Diabetes TubInt [34] p= 0.046, rs= -0.611). (B) The mRNA expression of IGHM was negatively correlated with GFR in 2 datasets: (a) Woroniecka Diabetes Glom GSE30528 [23] p= 0.003, rs= -0.604 and (b) Woroniecka Diabetes TubInt GSE30529 [23] p= 8.15e-5, rs= -0.740).
