# Supplementary material for: Complement Cascade Proteins Correlate with Fibrosis and Inflammation in Early-Stage Type 1 Diabetic Kidney Disease in the Ins2Akita Mouse Model
Source: Int J Mol Sci. 2024 Jan 23;25(3):1387. doi: 10.3390/ijms25031387 (PMC10855735; doi:10.3390/ijms25031387)
Supplement: Supplementary file 1 [file ijms-25-01387-s001.zip › Tables S1 and S2.pdf]

	Complement Cascade		
**fibrosis-related proteins**	**Ins2Akita 2 months**	**Ins2Akita 4 months**	**Ins2Akita 2&4months**
**ABAT**	**rs = 0.857, p = 0.007(C3), rs = 0.964, p = 0.0005 (IGHM),** rs=0.635, p=0.09 (C4B)	rs = 0.143, p= 0.736 (C3), rs = -0.371, p= 0.365 (C4B), rs = 0.036, p= 0.939 (IGHM)	**rs = 0.596, p= 0.019 (C3),** rs = 0.346, p = 0.189 (C4B), **rs = 0.632, p= 0.011 (IGHM)**
**FN1**	rs = -0.285, p= 0.534 (C3), rs = -0.558, p= 0.192 (C4B), rs = -0.485, p= 0.328 (IGHM)	**rs = 1, p = 0 (C3), rs = 0.761, p= 0.028 (C4B),** rs = 0.214, p= 0.610 (IGHM)	rs = 0.349, p= 0.221 (C3), rs = 0.473, p = 0.075 (C4B), **rs = 0.674, p= 0.008 (IGHM)**
**ARF6**	rs = -0.333, p= 0.419 (C3), rs = -0.059, p= 0.887 (C4B), rs = -0.75, p= 0.052 (IGHM)	rs = -0.464, p = 0.293 (C3), **rs = 0.738, p = 0.037 (C4B**), rs = 0.119, p = 0.778 (IGHM)	rs = -0.2, p = 0.475 (C3), rs = 0.278, p = 0.297 (C4B), rs = 0.021, p = 0.934 (IGHM)
**STXBP1**	rs = 0.2, p= 0.747 (C3), rs = 0.051, p= 0.934 (C4B), rs = 0.4, p= 0.6 (IGHM)	rs = -0.6, p = 0.4 (C3), **rs = 1, p = 0 (C4B),** rs = -0.8, p = 0.2 (IGHM)	rs = 0.55, p = 0.125 (C3), rs = 0.644, p = 0.061 (C4B), **rs = 0.714, p= 0.047 (IGHM)**
**FLNA**	rs = 0.428, p = 0.289 (C3), rs = 0.67, p= 0.068 (C4B), rs = 0.642, p= 0.119 (IGHM)	rs = 0.071, p = 0.879 (C3), **rs = 0.714 p = 0.047 (C4B),** rs = 0.095, p = 0.822 (IGHM)	**rs = 0.586, p = 0.022 (C3), rs = 0.79, p = 0.0002 (C4B), rs = 0.776, p = 0. 0004(IGHM),**
**CYFIP1**	rs = -0.642, p = 0.119 (C3), rs = -0.414, p= 0.355 (C4B), rs = -0.257, p = 0.622 (IGHM)	rs = 0.535, p = 0.215 (C3), **rs = 0.762, p = 0.028 (C4B),** rs = 0.428, p = 0.289 (IGHM)	rs = -0.147, p = 0.615 (C3), rs = 0.048, p = 0.864 (C4B), rs = 0.09, p = 0.76 (IGHM)
**MYOF**	**rs = 0.81, p = 0.015(C3), rs = 0.826, p = 0.011 (C4B), rs = 0.929, p= 0.002 (IGHM)**	rs = 0.142, p = 0.759 (C3), **rs = 0.905, p = 0.002 (C4B),** rs = 0.38, p = 0.352 (IGHM)	**rs = 0.603, p = 0.017(C3), rs = 0.892, p = 0 (C4B), rs = 0.786, p = 0.0005 (IGHM)**
**IQGAP1**	rs = -0.69, p = 0.058 (C3), rs = -0.071, p= 0.866 (C4B), rs = -0.357, p= 0.43 (IGHM)	rs = -0.178, p = 0.702 (C3), **rs = 0.714, p = 0.047 (C4B),** rs = 0.357, p = 0.385 (IGHM)	rs = -0.21, p= 0.451 (C3), rs = 0.21, p = 0.437 (C4B), rs = 0.235, p = 0.4 (IGHM)
**ITGB1**	rs = 0.19, p = 0.651 (C3), rs = 0.191, p = 0.649 (C4B), rs = 0.607, p = 0.148 (IGHM)	rs = 0.214, p = 0.645 (C3), **rs=0.69, p=0.058 (C4B),** rs = 0.452, p = 0.26 (IGHM)	rs = 0.467, p = 0.079 (C3), **rs = 0.596, p = 0.015 (C4B), rs = 0.675, p = 0.006 (IGHM)**
**ITGA1**	**rs = 0.762 p = 0.028 (C3**), rs = 0.61, p= 0.108 (C4B), **rs = 0.929, p = 0.003 (IGHM)**	rs = 0.178, p = 0.702 (C3), rs = 0.357, p = 0.385 (C4B), rs = 0.166, p = 0.693 (IGHM)	**rs = 0.664, p= 0.007(C3), rs = 0.57 p = 0.021(C4B), rs = 0.693, p = 0.004 (IGHM)**

Supplementary Table S1. Results of the Spearman rho correlation analysis of expression of the complement proteins vs fibrosis-related proteins performed in mice of 2-months, 4-months-old and the two groups combined. Statistically significant correlations are highlighted in green. The complement protein names with statistically significant correlation of expression with fibrosis-related proteins are shown into parentheses.

	Complement Cascade		
**inflammation-related proteins**	**Ins2Akita 2months**	**Ins2Akita 4months**	**Ins2Akita 2&4months**
**CD81**	rs = 0.428, p= 0.337 (C3), rs = 0.142, p= 0.759 (C4B), rs = 0.714, p= 0.11 (IGHM)	rs = 0.107, p= 0.819 (C3), rs = 0.214, p= 0.644 (C4B), rs = -0.178, p= 0.701 (IGHM)	**rs = 0.534, p= 0.049 (C3), rs = 0.56, p= 0.037 (C4B),** **rs = 0.742, p = 0.004 (IGHM)**
**ICAM1**	rs = -0.1, p= 0.872 (C3), rs = -0.1, p= 0.872 (C4B), rs = -0.8, p= 0.2 (IGHM)	rs = 0.6, p= 0.208 (C3), **rs = 0.857, p= 0.013 (C4B),** rs = 0.75, p= 0.052 (IGHM)	rs = 0.572, p = 0.066 (C3), **rs = 0.622 p= 0.031 (C4B), rs = 0.8, p= 0.003 (IGHM)**
**VNN1**	**rs = 0.9, p= 0.037 (C3), rs = 0.9, p= 0.037 (C4B),** rs = 0.8, p= 0.2 (IGHM)	rs = 0.3, p= 0.623 (C3), rs = -0.25714, p= 0.622 (C4B), rs = -0.25714, p= 0.622 (IGHM)	**rs = 0.745, p = 0.013 (C3)**, rs = 0.363, p= 0.272 (C4B), rs = 0.454, p= 0.187 (IGHM)
**SNAP23**	rs = 0.2619, p= 0.531 (C3), rs = 0.08383, p= 0.844 (C4B), **rs = 0.75, p= 0.05 (IGHM)**	rs = 0.286, p= 0.535 (C3), **rs =0.857, p=0.006 (C4B),** rs = 0.357, p= 0.385 (IGHM)	**rs = 0.568, p= 0.03 (C3), rs = 0.487, p= 0.05 (C4B), rs = 0.71, p= 0.003 (IGHM)**

**HSPD1**	**rs= 0.762, p= 0.028 (C3),** rs = 0.4551, p= 0.257 (C4B), **rs = 0.929, p= 0.003 (IGHM)**	rs = 0.214, p= 0.64 (C3), rs = -0.333, p= 0.42 (C4B), rs = 0.095, p= 0.823 (IGHM)	rs = 0.396, p= 0.143 (C3), rs = 0.115, p= 0.672 (C4B), rs = 0.204, p= 0.467 (IGHM)
**TSPAN2**	**rs = -0.9, p= 0.037 (C3), rs = -0.9, p= 0.037 (C4B),** rs = -0.8, p= 0.2 (IGHM)	rs = 0.142, p= 0.76 (C3), **rs = 0.762, p= 0.028 (C4B),** rs = 0.381, p= 0.352 (IGHM)	rs = -0.161, p= 0.618 (C3), rs = 0.269, p= 0.373 (C4B), rs = 0.328, p= 0.3 (IGHM)

Supplementary Table S2. Results of the Spearman rho correlation analysis of expression of the complement proteins vs inflammation-related proteins performed between in mice of 2-months, 4-months-old and the two groups combined. Statistically significant correlations are highlighted in green. Complement proteins with statistically significant correlation of expression with inflammation-related proteins are shown into parentheses.
